# Reducing the matrix effect in mass spectral imaging of biofilms using flow-cell culture

**DOI:** 10.3389/fchem.2023.1203314

**Published:** 2023-05-25

**Authors:** Yuchen Zhang, Andrew Plymale, Jiyoung Son, Qiaoyun Huang, Wenli Chen, Xiao-Ying Yu

**Affiliations:** ^1^ National Research Center for Edible Fungi Biotechnology and Engineering, Key Laboratory of Applied Mycological Resources and Utilization, Ministry of Agriculture, Shanghai Key Laboratory of Agricultural Genetics and Breeding, Institute of Edible Fungi, Shanghai Academy of Agricultural Sciences, Shanghai, China; ^2^ State Key Laboratory of Agricultural Microbiology, Huazhong Agricultural University, Wuhan, China; ^3^ Pacific Northwest National Laboratory, Energy and Environment Directorate, Richland, WA, United States; ^4^ Oak Ridge National Laboratory, Materials Science and Technology Division, Oak Ridge, TN, United States

**Keywords:** flow-cell culture, ToF-SIMS, biofilms, matrix effects, principal component analhysis (PCA)

## Abstract

The interactions between soil microorganisms and soil minerals play a crucial role in the formation and evolution of minerals and the stability of soil aggregates. Due to the heterogeneity and diversity of the soil environment, the under-standing of the functions of bacterial biofilms in soil minerals at the microscale is limited. A soil mineral-bacterial biofilm system was used as a model in this study, and it was analyzed by time-of-flight secondary ion mass spectrometry (ToF-SIMS) to acquire molecular level information. Static culture in multi-wells and dynamic flow-cell culture in microfluidics of biofilms were investigated. Our results show that more characteristic molecules of biofilms can be observed in SIMS spectra of the flow-cell culture. In contrast, biofilm signature peaks are buried under the mineral components in SIMS spectra in the static culture case. Spectral overlay was used in peak selection prior to performing Principal component analysis (PCA). Comparisons of the PCA results between the static and flow-cell culture show more pronounced molecular features and higher loadings of organic peaks of the dynamic cultured specimens. For example, fatty acids secreted from bacterial biofilm extracellular polymeric substance are likely to be responsible for biofilm dispersal due to mineral treatment up to 48 h. Such findings suggest that the use of microfluidic cells to dynamically culture biofilms be a more suitable method for reducing the matrix effect arisen from the growth medium and minerals as a perturbation fac-tor for improved spectral and multivariate analysis of complex mass spectral data in ToF-SIMS. These results show that the interaction mechanism between biofilms and soil minerals at the molecular level can be better studied using the flow-cell culture and advanced mass spectral imaging techniques like ToF-SIMS.

## 1 Introduction

Soil is dominated by a solid phase composed of particles of varying sizes and it is surrounded by the aqueous and gaseous phase (Stotzky, 1986). Clay minerals are important constituents of soils and they play an important role in environmental processes, such as nutrient cycling, plant growth, pollutant transport, organic matter maturation, and oil production (Dong et al., 2009). Soil microbiota, as the most active factor in soil, is involved in biogeochemical cycles, such as maintaining nutrient cycling, soil fertility, and carbon and nitrogen balance (Liang and Balser, 2011; Ahemad and Kibret, 2014) Microorganisms in soil often exist in the form of surface-attached structures known as biofilms (Flemming and Wingender, 2010; Flemming et al., 2016), which colonize on soil media like plant roots, clay minerals, carbon-rich surfaces; and they secrete extracellular polymeric substance (EPS) to form aggregates (Cai et al., 2019). The adsorption of soil biofilms on mineral surfaces plays a crucial role in mineral weathering, formation, and stabilization of soil aggregates, degradation and storage of organic carbon, and bioremediation of soil pollutants (Ma et al., 2017). Therefore, it is of great significance to study the formation of biofilm and its interactions with soil minerals.

The soil environment is dynamic and complex, and it poses difficulties in studying soil biofilms *in situ*. Hence, *ex situ* laboratory studies of representative strains are used to investigate soil biofilms. Clay minerals are hydrous aluminosilicate minerals with layered structures, mostly consisting of kaolinite, montmorillonite, and illite as main components of soil (Kittrick, 1969; Carrier and Kounaves, 2015). The chemical components of clay minerals are mainly silica, alumina and water (Weaver and Pollard, 2011). We use a mixture of silica, iron oxide, and alumina to simulate soil clay minerals. The strain selected in this work is a Gram-negative facultative anaerobic bacterium *Shewanella oneidensis* (Fredrickson et al., 2008). *S. oneidensis* MR-1 is remarkable in its anaerobic versatility and extracellular electron transfer ability (von Canstein et al., 2008; Shi et al., 2012). In the absence of oxygen and other electron acceptors, *S. oneidensis* MR-1 is able to utilize Fe(III) oxide minerals as terminal electron acceptors for anaerobic respiration (Mukherjee et al., 2020). Therefore, many researchers choose *S. oneidensis* MR-1 to study microbial-induced iron or manganese oxide mineral dissolution and associated biogeochemical cycling of soil microbiomes (Thormann et al., 2004; McLean et al., 2008; Ng et al., 2013; Ding et al., 2014; Zhao et al., 2015). In addition, choosing an appropriate and controllable cultivation method is crucial for the stable growth and utilization of *S. oneidensis* biofilms (Elias et al., 2008).

Various characterization techniques are used to study the interaction between biofilm and soil minerals. Recent studies expounded the selective adsorption characteristics and intrinsic mechanism of EPS components of *Pseudomonas putida* on different soil mineral surfaces (Lanni et al., 2014; Lin et al., 2016; Du et al., 2017; Ouyang et al., 2017; Qu et al., 2018). Confocal laser scanning microscopy (CLSM) imaging results showed that proteins in EPS were mainly distributed on the surface of montmorillonite and kaolinite; while nucleic acids were mainly distributed on the surface of goethite (Lin et al., 2016). The effect of soil minerals on the biofilm formation of the representative soil bacterium *Bacillus subtilis* was also explored. Atomic force microscopy (AFM) results showed the initial adaption process of *Bacillus subtilis* to soil minerals (Ma et al., 2017). Nuclear Magnetic Resonance spectroscopy and imaging were used to characterize *Shewanella oneidensis* MR-1 biofilms’ tomography, morphology, and metabolite products complemented with CLSM (Renslow et al., 2017). In this study, we use a very sensitive mass spectrometry technique ToF-SIMS (Time-of-Flight Secondary Ion Mass Spectrometry) for molecular characterization of biofilms. The unique advantage of ToF-SIMS analysis is that all mass signals within a specific mass range can be collected simultaneously, allowing detection of many surface compounds of interest with no labeling (Ding et al., 2016). However, data analysis of ToF-SIMS is challenging due to the big data nature and inherent matrix effects. The ionization process if ToF-SIMS is strongly influenced by changes in the solid surface chemistry and surface properties associated with the sputtering process, known as the matrix effect (Priebe et al., 2020). Matrix effects are often considered as a limiting factor in elemental characterization (Shishido et al., 2020). Secondary ion generation efficiencies can span five orders of magnitude or more due to matrix effects. This is the main reason for ToF-SIMS as a semi-quantitative technique (Priebe et al., 2020). Matrix effect also could raise difficulties in data interpretation (Ferrari and Ratner, 2000; Takahashi et al., 2017; Shishido et al., 2020), especially in complex biological samples (Jones et al., 2006). As to microbial samples, the influence of the matrix effect mainly comes from the rich nutrients and organic matter in the medium, because they contributed to organic interference peaks in the spectral analysis. When processing a large number of biological and microbial samples, the real differences between samples cannot be discerned, if peaks are not selected before performing the multivariant analysis (Yang et al., 2019). Therefore, it is critical to reduce the impact of matrix effects in ToF-SIMS analysis of biological specimens.

In this work, *S. oneidensis* MR-1 biofilms were cultured statically and dynamically using a microfluidics. The ToF-SIMS results obtained from different culture conditions were compared. We found that flow-cell culture is more conducive to effectively reduce the matrix effect in ToF-SIMS analysis. Thus, it is an improved approach to investigate soil mineral-bacterial biofilm interactions using ToF-SIMS in the future.

## 2 Materials and methods

### 2.1 Sample preparation of bacterial strain and mineral control samples

The *Shewanella oneidensis* MR-1 strain used in this study was purchased from ATCC. To prepare for the *S. oneidensis* MR-1 planktonic cell sample, a single colony was inoculated into 5 mL of tryptic soy broth (TSB) without dextrose medium (Marshall et al., 2008; McLean et al., 2008). TSB without dextrose medium is a low carbohydrate formulation, and the components include pancreatic digest of casein, enzymatic digest of soybean meal sodium chloride, and dipotassium phosphate. *S. oneidensis* MR-1 was incubated at 30°C and at 160 rpm for 12 h until the bacteria grew to the log phase and the OD_600_ reached about 1.6 (Supplementary Figure S1). Then, 1 mL of the bacterial-containing solution was added to a sterilized 1.5 mL centrifuge tube and centrifuged at 2500 rpm for 2 min. After centrifugation, the supernatant was discarded, and the strain was washed for three times with 1 mL of sterile deionized (DI) water. After discarding the supernatant for the last rinse, the strain was resuspended in 200 µL of sterile DI water. The samples were mixed with Vortex to obtain the desalinated planktonic cell control samples.

Silica, alumina, and iron oxide (ACS grades) were all purchased from Sigma-Aldrich and used as received. The reagents were mixed in a ratio of 5:1:0.5 to make 1 g/L simulated soil mineral suspension (Kittrick, 1969; Carrier and Kounaves, 2015). The *S. oneidensis* MR-1 planktonic cell and mineral mix control samples were deposited onto the clean silicon (Si) wafer (10 mm × 10 mm diced, Ted Pella Inc.) to prepare for samples for static ToF-SIMS analysis. All samples were completely dried in the biosafety cabinet before getting mounted onto the sample stage for analysis.

### 2.2 Static culture of biofilm

**FIGURE 1:**
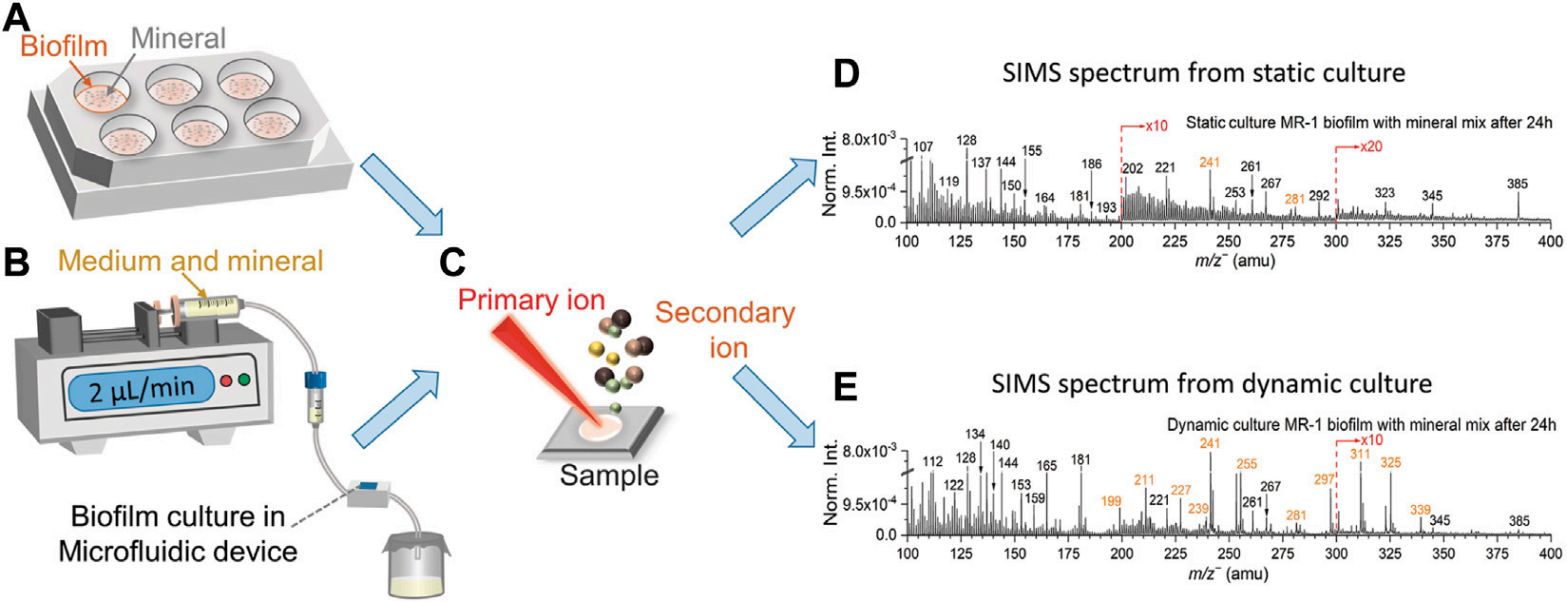
Biofilm static **(A)** and dynamic **(B)** culture setup, samples are collected on the Si wafers prior to ToF-SIMS analysis **(C)**. Dynamic **(D)** and static culture **(E)** spectral comparison of *S. oneidensis* MR-1 biofilm with mineral mix after 24 h in the negative mode in the range of *m/z* ^*−*^ 100–400. Fatty acids peaks are colored in orange.

A common feature of biofilm culturing methods is separating adherent bacteria from planktonic cells (Peterson et al., 2011; Azeredo et al., 2017). In this work, the static culture of *S. oneidensis* MR-1 biofilm was performed in 6-well cell culture plates (Figure 1A and Supplementary Figure S2A). 1 mL of log-phase bacterial solution was added into each well using a pipette, and plates were placed in the incubator at 30°C for biofilm static culture. During culture, 0.5 mL fresh medium was added every 48 h regularly to keep the bacteria alive. After 3–4 days, the light pink MR-1 biofilm was observed on the well surface (Supplementary Figure S2A), indicating that the biofilm was mature. The volume of culture was about 1 mL at this time. Then 1 mL of simulated soil mineral mix was then added to each well, and mixed well to homogenize. Samples were collected from each well at 4 h, 8 h, 12 h, 16 h, 24 h, and 48 h. The biofilm control sample was the sample without any interactions with the soil mineral mix simulant. 1.5 mL samples at each time point were pipetted into centrifuge tubes, and then quickly transferred to a ‒80°C freezer for storage. After all samples were collected, the samples were thawed, desalinated by centrifugation and washing, as indicated above, dried in the biosafety cabinet at room temperature, and then deposited onto clean Si wafers (Zhang et al., 2021) for ToF-SIMS analysis (Figure 1C).

### 2.3 Flow-cell dynamic culture of biofilms

The dynamic culture of *S. oneidensis* MR-1 biofilm was performed in a modified SALVI device (Figure 1B and Supplementary Figure S2B). Previous studies have described details of SALVI fabrication (Hua et al., 2014). The SALVI device was invented for probing liquid samples under high vacuum in ToF-SIMS and other instruments. Its microfluidic channel is bonded to a 100 nm thick silicon nitride (SiN) membrane by oxygen plasma. In this study, SiN membranes were replaced with clean silicon wafers. Compared with a SiN membrane, a Si wafer has the advantages of higher mechanical strength, lower fragility, and lower cost, making it ideal for culturing biofilm samples.

Sterilization, biofilm culture, and collection steps in microfluidic devices have been described in detail in previous studies (Komorek et al., 2017; Wei et al., 2017). Morphological information on the biofilms were captured in the SALVI device (Renslow et al., 2017). In this study, biofilm growth condition in the flow-cell device can be observed under the digital microscopy (Supplementary Figure S2C). After the biofilm was mature, biofilm control sample was collected without adding minerals. The TSB without dextrose medium was mixed with the mineral mix at a 1:1 volume ratio. For the mineral mix treated samples, the mixture of medium and minerals was filled into a 10 mL sterile syringe to continuously supply to the *S. oneidensis* MR-1 biofilm dynamic culture. A sterilized and sealed glass container placed at the end of the microfluidic culture system was used to collect the effluents at 4 h, 8 h, 12 h, 16 h, 24 h, and 48 h (Supplementary Figure S2B). The effluent samples desalinated, frozen, thawed, and dried in the same way as those obtained in static culture prior to static analysis in ToF-SIMS. The effluent sample contains the medium that has been continuously modified by the biofilms and the minerals. Thus, it can be used to reflect the biofilm activities as shown in the results. Samples were desalinated (Ding et al., 2016) and then deposited onto clean Si wafers (Zhang et al., 2021) for ToF-SIMS analysis (Figure 1C). Summary of the sample descriptions is shown in Supplementary Table S1.

### 2.4 ToF-SIMS

ToF-SIMS data were analyzed using the IONTOF Surface Lab 7.0 software and at least four positive and negative data points for each sample were collected. The ions used for mass calibration in the positive mode included CH_4_N^+^ (*m/z* ^*+*^ 30), C_3_H_9_N_2_^+^ (*m/z* ^*+*^ 73), CH_9_N_4_^+^ (*m/z* ^*+*^ 77), and C_19_H_39_O_2_^+^ (*m/z* ^*+*^ 299). The ions used for mass calibration in the negative mode were C^*−*^ (*m/z* ^*−*^ 12), C_4_H_7_O^*−*^ (*m/z* ^*−*^ 71), and C_20_H_39_O_2_^*−*^ (*m/z* ^*−*^ 311). The mass calibrated data were exported to Origin Pro (2019b) for plotting. Unit mass data were used for principal component analysis (PCA). They were pretreated by mean centering, normalization, and square root transformation before processing in Matlab (R2018b) (Graham et al., 2006).

## 3 Results and discussion

Figure 1B and Supplementary Figure S2B depict the dynamic setup, and a microfluidic cell was used to culture biofilm. The mineral component was mixed to the growth medium (TSB without dextrose medium) at a 1:1 volume ratio and used as nutrients to support the continued biofilm’s growth after the biofilms was formed in the growth chamber. Figures 1D, E show the SIMS spectral comparisons between the dynamic and static culture in the negative mode in the range of *m/z* ^−^ 100–400. The *S. oneidensis* MR-1 biofilms cultured using a medium solution and mineral mixture and collected 24 h after inoculation. Characteristic peaks of fatty acids associated with biofilms are colored in orange. Identification of these fatty acids is based on the high mass accuracy spectral results of the same strain and representative reference chemicals using static SIMS recently (Ding et al., 2016; Komorek et al., 2017). Detailed peak assignment in the negative mode is summarized in Supplementary Table S2.

The *S. oneidensis* MR-1 biofilm is formed by planktonic cells adhering to the substrate surface and their releasing EPS to encapsulate themselves to form a larger colony. Fatty acids and lipids are key components of EPS (Ding et al., 2016; Komorek et al., 2017). Fatty acids peaks were easily observed from the effluents of the microfluidics flow-cell culture in this work. Prominent fatty acid peaks are mainly detected in the range of *m/z* ^*−*^ ≥200, such as myristic acid *m/z* ^*−*^ 227.24, C_14_H_27_O_2_^*−*^, pentadecanoic acid *m/z* ^*−*^ 241.26, C_15_H_29_O_2_^*−*^, palmitic acid *m/z* ^*−*^ 255.28, C_16_H_31_O_2_^*−*^, non-adecanoic acid *m/z* ^*−*^ 297.21, C_19_H_37_O_2_^*−*^, arachidic acid *m/z* ^*−*^ 311.23, C_20_H_39_O_2_^*−*^ and heneicosanoic acid *m/z* ^*−*^ 325.25, C_21_H_41_O_2_^*−*^. In addition, characteristic biofilm peaks observed in the same *m/z* range from the dynamic culture results have significantly higher abundance than those from the static culture. The same is true after normalization to the total ion intensities. In contrast, characteristic signals of fatty acids are buried under mineral or other inorganic components and could not be detected easily in the static cultured specimens.

**FIGURE 2:**
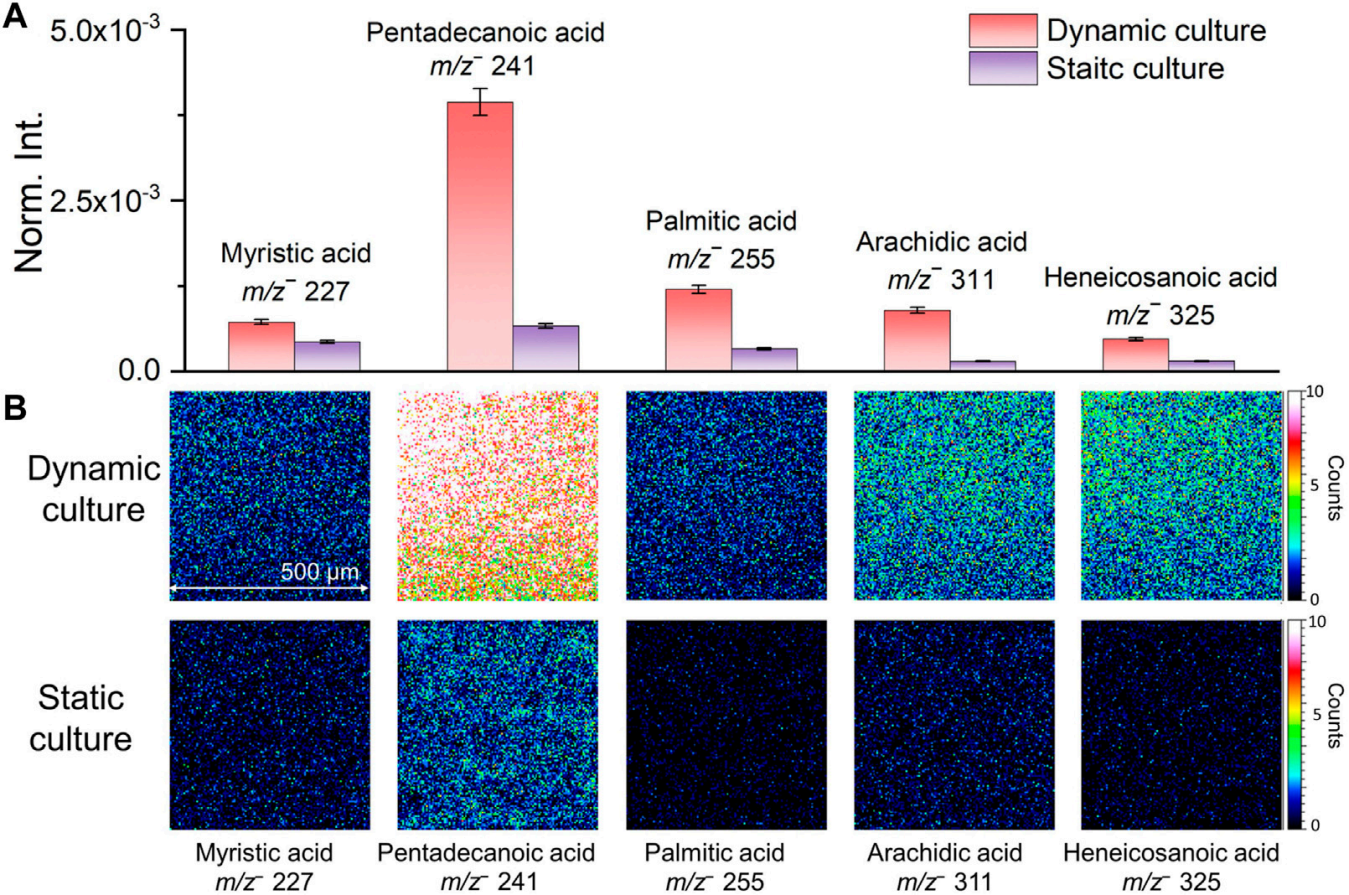
Comparison of normalized intensities **(A)** and 2D molecular imaging **(B)** of fatty acids under different biofilm culture methods.

Several representative fatty acid compounds were chosen as examples to show differences of characteristic biofilm peaks acquired from the two culturing methods intuitively. Figure 2A shows the normalized comparison of selected peaks in bar plots. Fatty acids peak intensities were higher in the flow culture shown in red than static culture shown in purple. The biggest difference was seen in pentadecanoic acid (*m/z* ^*−*^ 241.26, C_15_H_29_O_2_^*−*^). Figure 2B shows the 2D molecular imaging comparison results corresponding to these compounds in the dynamic and static culture. Darker colors correspond to lower ion intensities and lighter colors high ion intensities. Our results show that the normalized spectral and 2D image comparisons are consistent. Both indicated that the signal intensities of fatty acids in the dynamic culture were higher, making it more conducive to detecting characteristic biofilm peaks. The spectral and 2D image comparison results and associated peaks of the two biofilm culture methods suggest that microfluidic flow cell culture of biofilms should be more suitable to study the interfacial reactions between *S. oneidensis* MR-1 biofilms and soil minerals. This finding is consistent with previous reports that flow cell has advantages in biofilm culture (Yang et al., 2013; Yu et al., 2013).

**FIGURE 3:**
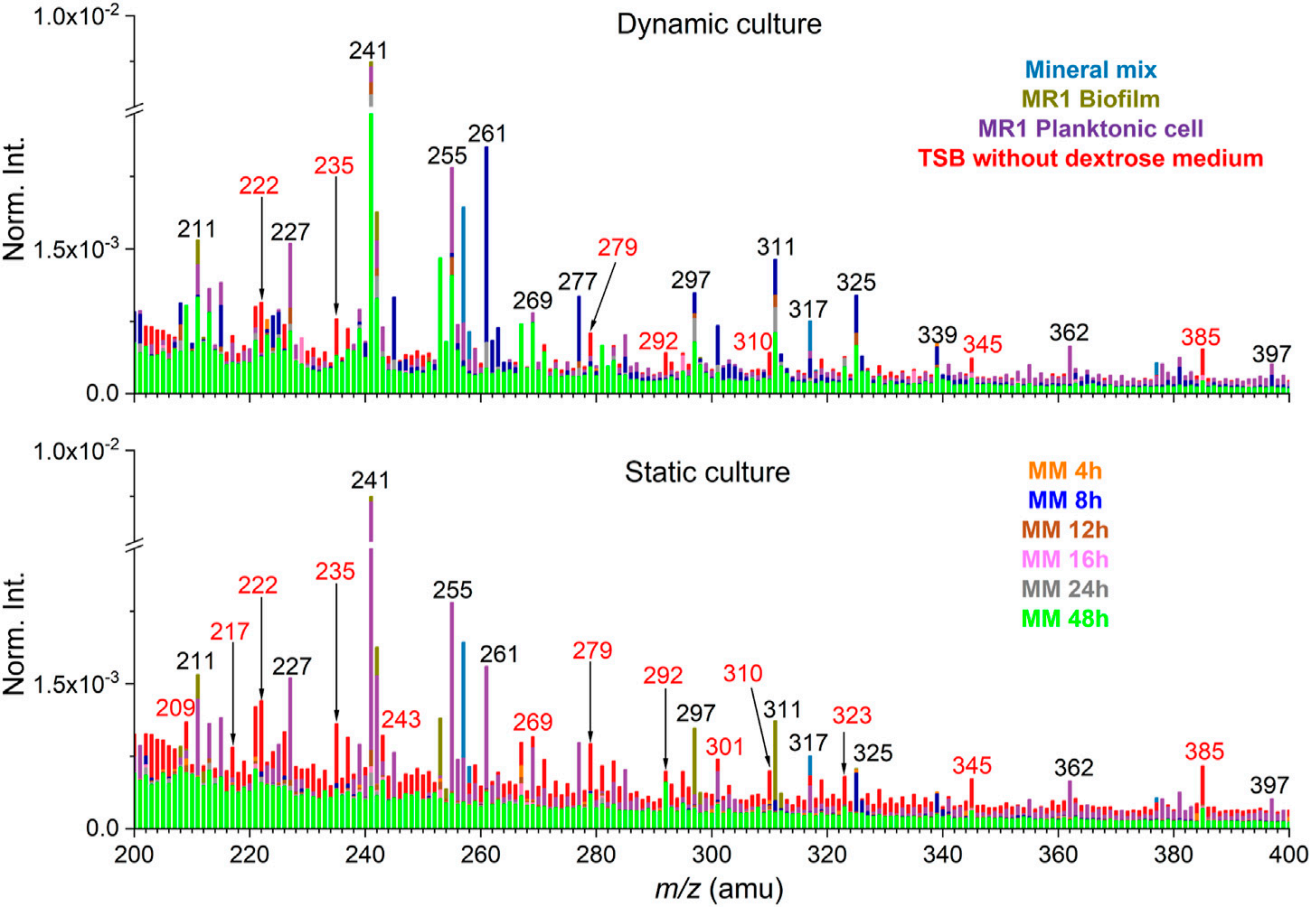
Spectral overlay comparison between dynamic and static cultures in the negative mode (*m/z* ^*−*^ 200–400). Interfering peaks from the medium solution are colored in red. “MM” stands for the mineral mix and *S. oneidensis* MR-1 biofilm mixtures collected as effluents.

Additionally, PCA was performed of the SIMS spectral data from the biofilm and soil minerals system under the static and dynamic culture condition. Spectral overlay was used to select peaks and to potentially reduce the matrix effect (Yang et al., 2019). Partial SIMS spectral overlay comparison results between static and dynamic setup of the negative mode is shown in Figure 3 and Supplementary Figure S3. Interference peaks from the medium solution are colored in red. The spectral overlay comparison results of *m/z* ^−^ 200–400 show that interference peaks are not significant in the dynamic setup. This is an interesting finding. As shown by the result of the static culture, the interference peaks from the medium solution are known to have high presence in this spectral range (Yang et al., 2019). This is likely attributed to the flow-cell culture setup, where biofilm peaks were prominent and not buried by high abundance inorganic ions or other organics in the matrix, reducing the matrix effect. Specifically, inorganic ion peaks from mineral components are mostly distributed in the range of *m/z* ^−^ ≤100 and dominate in this range, for instance, silica (*m/z* ^*−*^ 59.97, SiO_2_^*−*^) and its fragment (*m/z* ^*−*^ 76.98, SiHO_3_^*−*^). Although the matrix effect of the flow-cell biofilm sample set was not significant in this work, additional interfering peaks from the medium could still be removed using the spectral overlay method. Furthermore, it is necessary to select peaks according to the spectral comparison and all peak PCA results to put an emphasis on the contribution of characteristic peaks. This approach could reduce the matrix effects arisen from interferences other than the medium solution (Ding et al., 2016).

**FIGURE 4:**
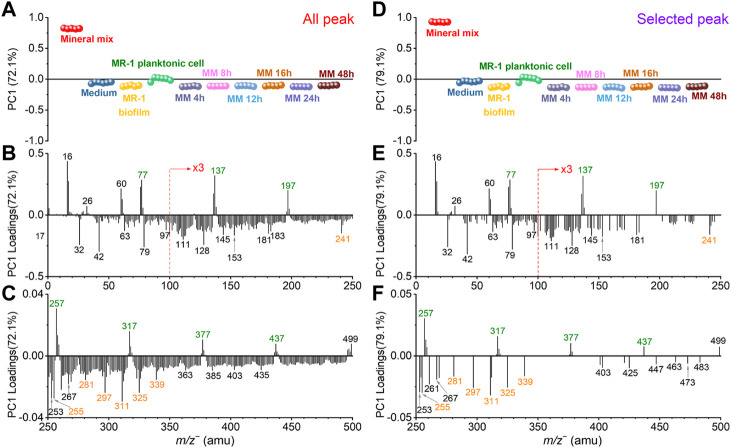
Comparison of all peak and selected peak PC1 results in the negative mode. All peak PC1 scores **(A)** and loadings plots in the *m/z* ^*−*^ range of 0–250 **(B)** and 250–500 **(C)**, respectively. Similarly, selected peak PC1 scores **(D)** and loadings plots in the *m/z* range of 0–250 **(E)** and 250–500 **(F)**, respectively. Mineral peaks and fatty acid peaks are marked in green and orange for ease of viewing.

Comparison results of SIMS all peak (*m/z* ^−^ 0–800.) and selected peak spectral PCA are shown in Supplementary Figure S3 and Figure 4. The biofilm characteristic peaks and medium interference peaks are colored in blue and orange, respectively. Since the overall ion intensity of the mass spectral peaks in the *m/z* ^*‒*^ 550–800 range is relatively low, the PCA results discussed here focus on the *m/z* ^‒^ 0–550 range.

Figure 4 shows the SIMS spectral PCA comparison results regarding PC1 in the negative mode. Figure 4A depicts all peak PC1 scores result. PC1 explains 72.1% of all data, and it distinguishes the mineral mix from other samples. Figure 4D shows selected peak spectral PCA score result. PC1 explains 79.6% of all data, and the distinction among samples is consistent with all peak PC1. The scores results of these PCA show that: 1) PC1 can separate mineral oxide mixtures from organic matter-based biological biofilms; 2) After the *S. oneidensis* MR-1 biofilm is exposed to mineral oxides, there is no difference in the distribution of microbial induced products at different time points from PC1. These samples are clustered in the negative PC1, and they are closer to the *S. oneidensis* MR-1 biofilm control sample.

The scores value of medium and other samples did not change significantly in the selected peak PCA results. This finding indicates that the interference peaks from the medium solution in PC1 are not the main reason for the differences in the samples, suggesting that the matrix effect is reduced. Many peaks have low loadings in the negative region of all peak PC1 in Figures 4B, C. They may come from system noise or incompletely removed salts in the microbial samples. These peaks do not affect the loadings of the biofilm relevant peaks. The mineral oxide peaks with positive loadings and the biofilm peaks located at the negative loadings of PC1 still have significant contributions. The selected peak PC1 results (Figures 4D–F) also further demonstrate that there is no significant gain between the scores and loadings results after peak selection based on spectral overlay. Combined with the score results, we find that several peaks, such as SiHO_3_^*−*^ (*m/z* ^*−*^ 76.98), Si_2_HO_5_^*−*^ (*m/z* ^*−*^ 136.94), Si_3_HO_7_^*−*^ (*m/z* ^*−*^ 196.91), Si_4_HO_9_^*−*^ (*m/z* ^*−*^ 256.88), Si_5_HO_11_^*−*^ (*m/z* ^*−*^ 316.85), Si_6_HO_13_^*−*^ (*m/z* ^*−*^ 376.81) and Si_7_HO_15_^*−*^ (*m/z* ^*−*^ 436.79), are main components or products of the mineral mix, and interesting the mass difference may be related to the addition of silica (*m/z* ^*−*^ 59.97, SiO_2_^*−*^) as one of the main components of the simulated soil. This finding also suggests that clustering.

**FIGURE 5:**
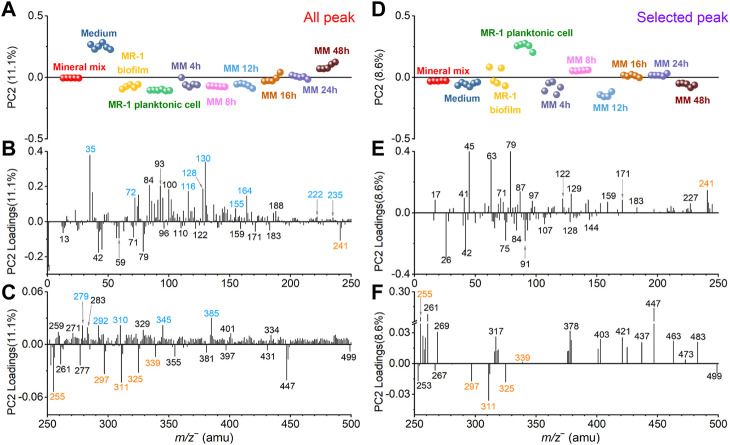
Comparison of all peak and selected peak PC2 results in the negative mode. All peak PC2 scores **(A)** and loadings plots in the *m/z* ^*−*^ range of 0–250 **(B)** and 250–500 **(C)**, respectively. Similarly, selected peak PC2 scores **(D)** and loadings plots in the *m/z* range of 0–250 **(E)** and 250–500 **(F)**, respectively. Fatty acid and medium interference peaks are marked in orange and blue, respectively, for ease of viewing.

Similarly, Figure 5 shows the spectral PCA comparison results regarding PC2 in the negative mode. Figure 5A depicts the all-peak PC2 scores plot. PC2 explains 11.1% of all data. The scores plot shows that the medium sample has the highest value in the positive PC2. The *S. oneidensis* MR-1 biofilm and effluents collected after adding the mineral mix into the medium solution after 4 h, 8 h, and 12 h are closer to the *S. oneidensis* MR-1 biofilm and planktonic cell control samples. The 16 h and 24 h samples gradually approach to the mineral mix, and the 48 h sample is even closer to the medium sample. In Figure 5D, the selected peak PC2 explains 8.6% of all data. After removing peaks according to the spectral overlay and running PCA, the PC2 scores change. The value of the medium sample has decreased and located in the negative PC2 region. The clustering of the other samples changes too. The 4 h sample is close to the biofilm control, and the 8 h sample is between the planktonic cells and the biofilm control. However, the 12 h sample lies in negative PC2. The 16 h and 24 h samples are between the mineral mix and the biofilm control. The 48 h sample is closer to the biofilm control and medium. These results of PC2 indicate temporal differences after the *S. oneidensis* MR-1 biofilm is exposed to the mineral mixture as part of the nutrient. We analyze possible reasons of the observed dynamic changes in conjunction with the PCA loadings results.

In the positive loadings of all peak PC2, peaks from the medium solution marked in blue color, such as Cl^*−*^ (*m/z* ^*−*^ 34.97), C_9_H_8_^*−*^ (*m/z* ^*−*^ 116.06), C_6_H_12_NO_2_^*−*^ (*m/z* ^*−*^ 130.09), C_9_H_10_NO_2_^*−*^ (*m/z* ^*−*^ 164.07), and C_3_HO_7_Na_7_^*−*^ (*m/z* ^*−*^ 309.98), make major contributions (Figures 5B, C), leading to the largest value of the medium in the scores (Figure 5A). After removing these interfering peaks, the selected peak PC2 loadings results (Figures 5E, F) illustrate the contributions of biofilm characteristic peaks more readily.

For example, pentadecanoic acid (*m/z* ^*−*^ 241.26, C_15_H_29_O_2_^*−*^) and palmitic acid (*m/z* ^*−*^ 255.28, C_16_H_31_O_2_^*−*^) are important contributors in the positive loadings of selected peak PC2. Combined with the scores result (Figure 5D), these two fatty acid peaks are mainly from the 8 h and planktonic cell samples. The 8 h effluent sample has the highest positive score value, and its composition is the closest to the planktonic cell, suggesting that the *S. oneidensis* MR-1 biofilm partially disperse to the planktonic state under the influence of minerals at this time. Previous studies have shown that exposure of *S. oneidensis* MR-1 living biofilms to the heavy metal ions Cr VI) leads to biofilm dispersal, and this process is mediated by palmitic acid. With the addition of Cr VI), the palmitic acid content increases, thereby causing the dispersal of biofilms (Ding et al., 2016; Ding et al., 2019). This work shows that the addition of mineral oxides could induce the synthesis of palmitic acid, which leads to biofilm dispersal. Pentadecanoic acid (*m/z* ^*−*^ 241.26, C_15_H_29_O_2_^*−*^) also plays a role in this process. In addition, docosanoic acid (*m/z* ^*−*^ 339.27, C_22_H_43_O_2_^*−*^) may be involved, although its contribution is not as high. The 12 h sample is distinct from all other samples according to the PC2 scores plot (Figure 5D). Our SIMS observations and data analysis suggest that the *S. oneidensis* MR-1 biofilm is most affected by the mineral mixture and its composition changes significantly after 12-h exposure. Non-adecanoic acid (*m/z* ^*−*^ 297.21, C_19_H_37_O_2_^*−*^), arachidic acid (*m/z* ^*−*^ 311.23, C_20_H_39_O_2_^*−*^), and heneicosanoic acid (*m/z* ^*−*^ 325.25, C_21_H_41_O_2_^*−*^) are the main contributors to this process when looking into the loadings results in Figures 5E, F.

In this study, we used ToF-SIMS and dynamic culture to explore the interfacial interaction mechanism between the *S. oneidensis* MR-1 biofilm and a simulated soil mineral mixture. The spectral PCA results show that the composition of the biofilm has temporal changes after the MR-1 biofilm is exposed to soil minerals. Several fatty acids (i.e., palmitic acid, pentadecanoic acid, arachidic acid) exhibit strong effects in this dynamic process. It is postulated that they also are responsible for biofilm dispersal. These fatty acids are integral components of the biofilm EPS, indicating the role of EPS components in the interactions of soil minerals.

## 4 Conclusion

In conclusion, we demonstrate dynamic flow cell culture approach using microfluidic devices is suitable for preparing microbial biofilms for ToF-SIMS analysis. Spectral and molecular imaging comparison results show that more biofilm characteristic peaks could be observed in the dynamic culture condition. In contrast, biofilm signals acquired from the static culture conditions are buried by minerals or other non-biofilm components. This finding suggests that sample preparation is critical in microanalysis and molecular imaging of bacterial biofilms, especially in sensitive surface analysis techniques such as ToF-SIMS. The microfluidic growth chamber is flexible in microbial culture and media tuning, both are important in simulating a variety of conditions to understand microbes and soil interactions at the microscale.

We analyzed *S. oneidensis* MR-1 biofilm cultured using the dynamic method. We used spectral overlay as a peak selection strategy to explore the reaction products between *the* MR-1 biofilm and simulated soil mineral mixture. The PCA results show that the compositional changes of the minerals and the MR-1 biofilm have time dependence. After 8-h of exposure to minerals, the biofilm disperses under the influence of palmitic acid and pentadecanoic acid. After 12-h, the biofilm is further damaged likely under the effect of arachidic acid and other fatty acids. To study the biofilm−soil mineral interactions, ToF-SIMS offers full spectral information of the individual components of biofilm EPS such as polysaccharides and proteins as well as mineral oxide makeups. Our findings not only provide molecular evidence of the interaction between biofilms and soil minerals, but also expand the application of ToF-SIMS in microbiology and systems biology.

## Data Availability

The original contributions presented in the study are included in the article/Supplementary Material, further inquiries can be directed to the corresponding author.

## Licenses and permissions

This manuscript has been authored by UT-Battelle, LLC under Contract No. DE-AC05-00OR22725 with the U.S. Department of Energy. The United States Government retains and the publisher, by accepting the article for publication, acknowledges that the United States Government retains a non-exclusive, paid-up, irrevocable, world-wide license to publish or reproduce the published form of this manuscript, or allow others to do so, for United States Government purposes.
