# Supplementary material for: Reducing the matrix effect in mass spectral imaging of biofilms using flow-cell culture
Source: Front Chem. 2023 May 25;11:1203314. doi: 10.3389/fchem.2023.1203314 (PMC10248399; doi:10.3389/fchem.2023.1203314)
Supplement: Supplementary file 1 [file DataSheet1.docx]

Supplementary Material

Yuchen Zhang^1,2^, Andrew Plymale^3^, Jiyoung Son^3^, Qiaoyun Huang^2^, Wenli Chen^2^, and Xiao-Ying Yu^4^*

^1^National Research Center for Edible Fungi Biotechnology and Engineering, Key Laboratory of Applied Mycological Resources and Utilization, Ministry of Agriculture, Shanghai Key Laboratory of Agricultural Genetics and Breeding, Institute of Edible Fungi, Shanghai Academy of Agricultural Sciences, Shanghai, 201403, China

^2^State Key Laboratory of Agricultural Microbiology, Huazhong Agricultural University, Wuhan, 430070, China

^3^Energy and Environment Directorate, Pacific Northwest National Laboratory, Richland, WA, 99352, USA

^4^Materials Science and Technology Division, Oak Ridge National Laboratory, Oak Ridge, TN, 37830, USA

*** Correspondence:**Xiao-Ying Yu, email: [yuxiaoying@ornl.gov](mailto:yuxiaoying@ornl.gov)

Table of Contents

[Supplemental Figures S-3](#_Toc134361063)

[**Figure S1**. The growth curve of *Shewanella oneidensis* MR-1 acquired at 600 nm using the TSB without dextrose medium. S-3](#_Toc134361064)

[**Figure S2.** Photos showing the static (a) and dynamic flow-cell (b) culture setup of *Shewanella oneidensis* MR-1 biofilms. (c) The biofilm growth in the flow-cell device is observed under the digital microscopy. (d) The clean as fabricated microchannel before biofilm culture. S-4](#_Toc134361065)

[**Figure S3.** Spectral overlay comparison between dynamic and static setup in the negative mode (*m/z^−^* 0 – 200). Interfering peaks from the medium solution are colored in red. “MM” stands for the mineral mix and *S. oneidensis* MR-1 biofilm mixtures collected as effluents. S-5](#_Toc134361066)

[Supplemental Tables S-6](#_Toc134361067)

[**Table S1**. Summary of the sample descriptions S-6](#_Toc134361068)

[**Table S2**. Possible peak identification in the negative ion mode S-8](#_Toc134361069)

[References S-9](#_Toc134361070)

# Supplemental Figures


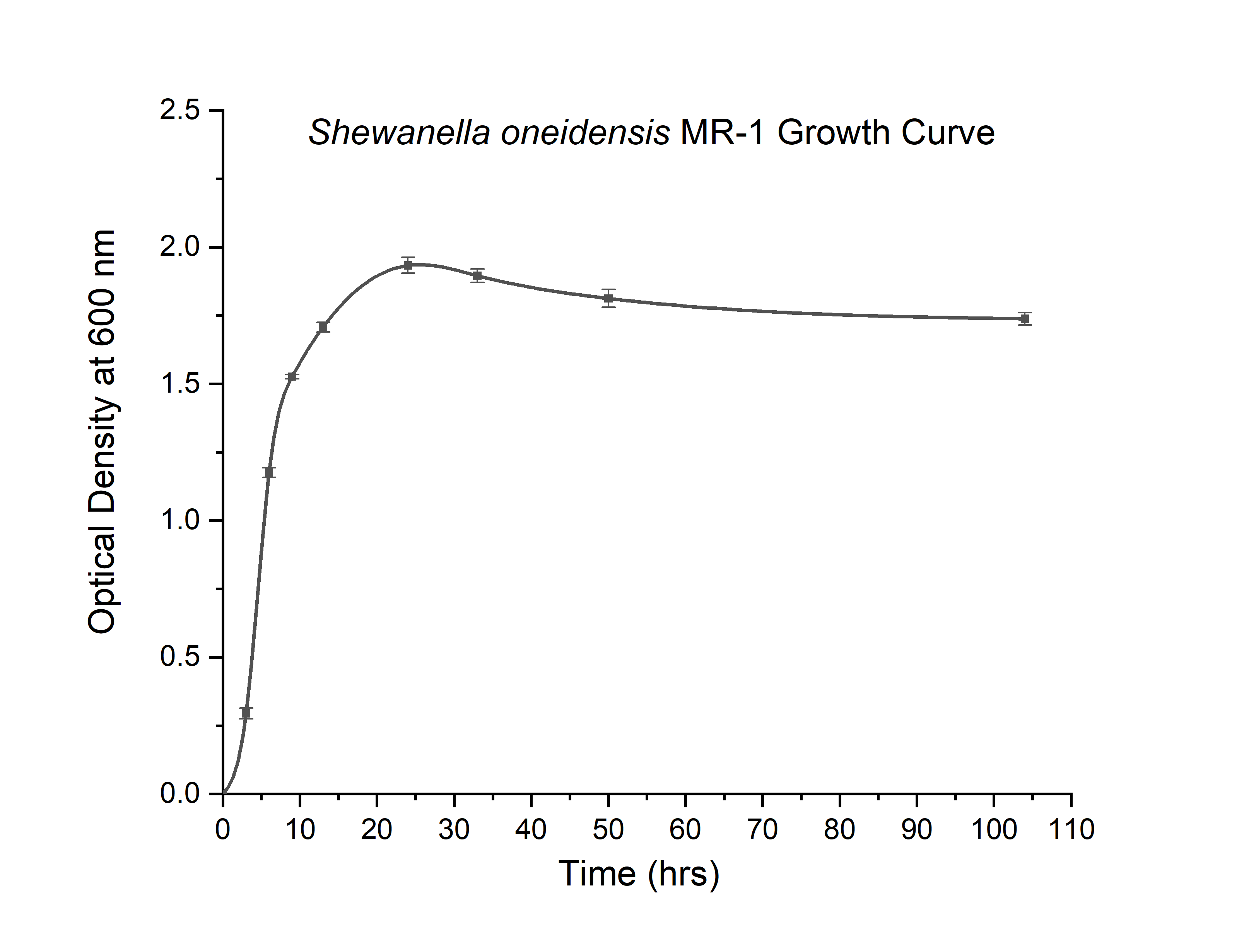


**Figure S1**. The growth curve of *Shewanella oneidensis* MR-1 acquired at 600 nm using the TSB without dextrose medium.


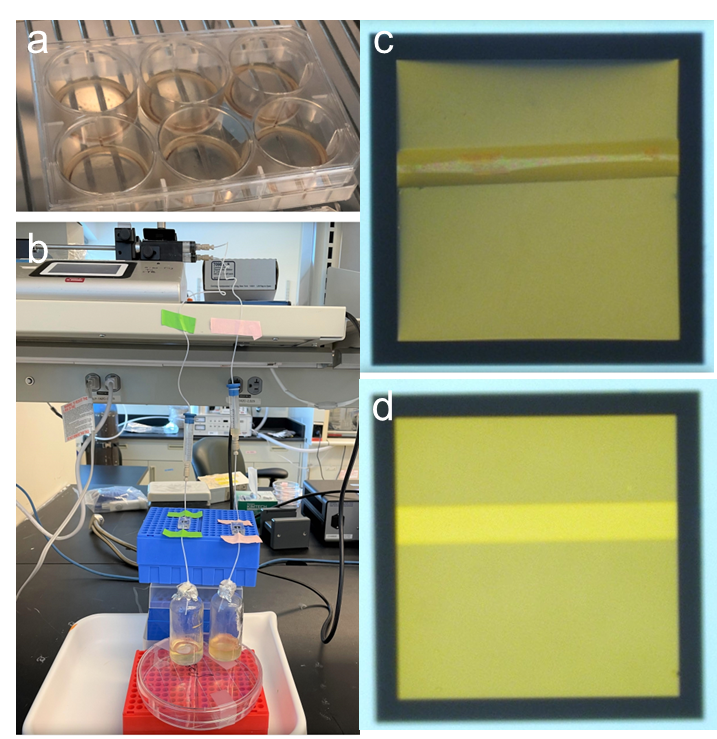


**Figure S2.** Photos showing the static (a) and dynamic flow-cell (b) culture setup of *Shewanella oneidensis* MR-1 biofilms. (c) The biofilm growth in the flow-cell device is observed under the digital microscopy. (d) The clean as fabricated microchannel before biofilm culture.


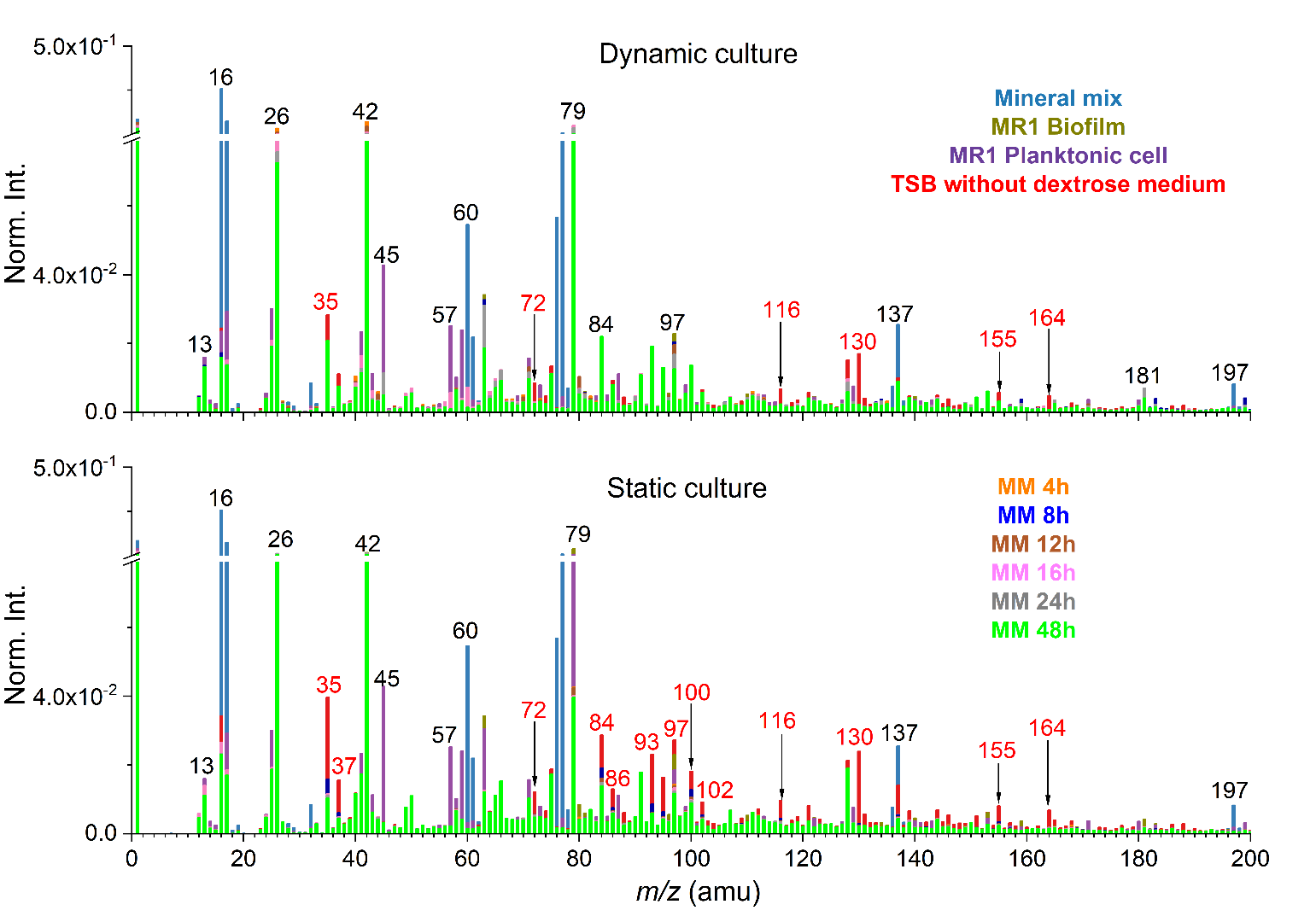


**Figure S3.** Spectral overlay comparison between dynamic and static setup in the negative mode (*m/z^−^* 0 – 200). Interfering peaks from the medium solution are colored in red. “MM” stands for the mineral mix and *S. oneidensis* MR-1 biofilm mixtures collected as effluents.

# Supplemental Tables

**Table S1**. Summary of the sample descriptions

| Sample | Brief name | Chemical details/ Preparation process | |
| --- | --- | --- | --- |
| Mineral mix and *S. oneidensis* MR-1 biofilm mixtures collected after 4 h | MM 4h | | Static culture: *S. oneidensis* MR-1 biofilm was performed in 6-well cell culture plates (Figure S2a). Samples were prepared as described in the main text.  Dynamic culture: *S. oneidensis* MR-1 biofilm was performed in a modified SALVI device (Figure S2b). Samples were prepared as described in the main text. |
| Mineral mix and *S. oneidensis* MR-1 biofilm mixtures collected after 8 h | MM 8h | | The procedure is the same as the “MM 4h” samples, which were collected at 8 h. |
| Mineral mix and *S. oneidensis* MR-1 biofilm mixtures collected after 12 h | MM 12h | | The procedure is the same as the “MM 4h” samples, which were collected at 12 h. |
| Mineral mix and *S. oneidensis* MR-1 biofilm mixtures collected after 16 h | MM 16h | | The procedure is the same as the “MM 4h” samples, which were collected at 16 h. |
| Mineral mix and *S. oneidensis* MR-1 biofilm mixtures collected after 24 h | MM 24h | | The procedure is the same as the “MM 4h” samples, which were collected at 24 h. |
| Mineral mix and *S. oneidensis* MR-1 biofilm mixtures collected after 48 h | MM 48h | | The procedure is the same as the “MM 4h” samples, which were collected at 48 h. |
| *S. oneidensis* MR-1 biofilm | MR-1 biofilm | | The procedure is the same as the “MM 4h” samples, which were collected at 0 h without any interactions with the soil mineral mix simulant. |
| *S. oneidensis* MR-1 planktonic cell | MR-1 planktonic cell | | A single *S. oneidensis* MR-1 colony was inoculated into 5 mL of TSB without dextrose medium and incubated at 30 ℃ and at 160 rpm for 12 hours until the bacteria grew to the log phase and the OD_600_ reached about 1.6. |
| TSB medium without dextrose | Medium | | Per liter medium contains casein enzymic hydrolysate 17.0 g, papaic digest of soybean meal 3.0g, sodium chloride 5.0g and dipotassium phosphate 2.5 g. |
| Mineral mix | Mineral mix | | Simulative mineral contains silica, alumina, and iron oxide (ACS grades). The reagents were mixed in a ratio of 5:1:0.5 to make 1 g/L simulated soil mineral suspension ([Kittrick 1969](#_ENREF_3); [Carrier and Kounaves 2015](#_ENREF_1)). |

**Table S2**. Possible peak identification in the negative ion mode

| *m/z*^−^_obs_ | *m/z*^−^_the_ | Δm/m  (ppm) | Formula | Chemical  descriptions | References |
| --- | --- | --- | --- | --- | --- |
| 34.971 | 34.969 | 30.5 | Cl*^−^* | Medium | ([Wei et al., 2017](#_ENREF_5)) |
| 59.969 | 59.967 | 20.5 | SiO_2_*^−^* | Silica | ([Wei et al., 2017](#_ENREF_5)) |
| 76.976 | 76.970 | 77.5 | SiHO_3_*^−^* | Silica | ([Ding et al., 2016](#_ENREF_2)) |
| 116.064 | 116.063 | 7.7 | C_9_H_8_*^−^* | Medium | This work |
| 130.090 | 130.087 | 17.8 | C_6_H_12_NO_2_*^−^* | Medium | This work |
| 136.931 | 136.937 | 42.4 | Si_2_HO_5_*^−^* | Silica | ([Zhang et al., 2021](#_ENREF_6)) |
| 164.073 | 164.072 | 7.8 | C_9_H_10_NO_2_*^−^* | Medium | This work |
| 196.902 | 196.904 | 9.2 | Si_3_HO_7_*^−^* | Silica | ([Zhang et al., 2021](#_ENREF_6)) |
| 199.169 | 199.170 | 3.6 | C_12_H_23_O_2_^−^ | Lauric acid | ([Ding et al., 2016](#_ENREF_2)) |
| 211.179 | 211.170 | 39.2 | C_13_H_23_O_2_^−^ | Fatty acid | ([Ding et al., 2016](#_ENREF_2)) |
| 227.205 | 227.201 | 16.1 | C_14_H_27_O_2_^−^ | Myristic acid | ([Ding et al., 2016](#_ENREF_2)) |
| 239.222 | 239.238 | 66.9 | C_16_H_31_O^−^ | Fatty acid | ([Ding et al., 2016](#_ENREF_2)) |
| 241.218 | 241.217 | 4.3 | C_15_H_29_O_2_^−^ | Pentadecanoic acid | ([Ding et al., 2016](#_ENREF_2)) |
| 255.233 | 255.232 | 1.3 | C_16_H_31_O_2_^−^ | Palmitic acid | ([Ding et al., 2016](#_ENREF_2); [Komorek et al., 2017](#_ENREF_4)) |
| 256.869 | 256.870 | 5.3 | Si_4_HO_9_*^−^* | Silica | ([Zhang et al., 2021](#_ENREF_6)) |
| 281.249 | 281.249 | 0.3 | C_18_H_33_O_2_^−^ | Oleic acid | ([Zhang et al., 2021](#_ENREF_6)) |
| 297.274 | 297.279 | 20.4 | C_19_H_37_O_2_^−^ | Nonadecanoic acid | ([Ding et al., 2016](#_ENREF_2)) |
| 309.908 | 309.901 | 20.1 | C_3_HO_7_Na_7_*^−^* | Medium | This work |
| 311.296 | 311.295 | 0.8 | C_20_H_39_O_2_^−^ | Arachidic acid | ([Ding et al., 2016](#_ENREF_2)) |
| 316.837 | 316.837 | 1.5 | Si_5_HO_11_*^−^* | Silica | This work |
| 325.317 | 325.311 | 17.7 | C_21_H_41_O_2_^−^ | Heneicosanoic acid | ([Ding et al., 2016](#_ENREF_2); [Komorek et al., 2017](#_ENREF_4)) |
| 339.338 | 339.326 | 32.3 | C_22_H_43_O_2_^−^ | Docosanoic acid | ([Ding et al., 2016](#_ENREF_2)) |
| 376.800 | 376.803 | 9.3 | Si_6_HO_13_*^−^* | Silica | This work |
| 436.762 | 436.770 | 19.5 | Si_7_HO_15_*^−^* | Silica | This work |

Note: *m/z*^−^_the_ represent the theoretical mass to charge ratio, *m/z*^−^_obs_ represent the observed mass to charge ratio.

# References

Carrier, B. L. and Kounaves, S. P. (2015). The origins of perchlorate in the martian soil. *Geophys. Res. Lett.* 42(10): 3739-3745. doi: 10.1002/2015GL064290

Ding, Y. Z., Zhou, Y. F., Yao, J., Szymanski, C., Fredrickson, J., Shi, L., et al. (2016). In situ molecular imaging of the biofilm and its matrix. *Anal. Chem.* 88(22): 11244-11252. doi: 10.1021/acs.analchem.6b03909

Kittrick, J. A. (1969). Soil minerals in al_2_o_3_-sio_2_-h_2_o system and a theory of their formation. *Clays Clay Miner.* 17(3): 157-167. doi: 10.1346/CCMN.1969.0170304

Komorek, R., Wei, W., Yu, X., Hill, E., Yao, J., Zhu, Z., et al. (2017). In situ characterization of Shewanella oneidensis MR1 biofilms by SALVI and ToF-SIMS. *J. Vis. Exp.*(126): e55944. doi: 10.3791/55944

Wei, W., Zhang, Y., Komorek, R., Plymale, A., Yu, R., Wang, B., et al. (2017). Characterization of syntrophic geobacter communities using ToF-SIMS. *Biointerphases* 12(5): 05G601. doi: 10.1116/1.4986832

Zhang, Y., Komorek, R., Son, J., Riechers, S., Zhu, Z., Jansson, J., et al. (2021). Molecular imaging of plant-microbe interactions on the brachypodium seed surface. *Analyst* 146(19): 5855-5865. doi: 10.1039/d1an00205h
